# Radial glia progenitor polarity in health and disease

**DOI:** 10.3389/fcell.2024.1478283

**Published:** 2024-10-02

**Authors:** Valeria Viola, Kaviya Chinnappa, Fiona Francis

**Affiliations:** ^1^ Institut du Fer à Moulin, Paris, France; ^2^ Institut National de Santé et de Recherche Médicale (INSERM, UMR-S 1270), Paris, France; ^3^ Faculty of Science and Engineering, Sorbonne University, Paris, France

**Keywords:** cortical development, cortical malformations, proliferation, neuronal migration, local translation, organelles

## Abstract

Radial glia (RG) are the main progenitor cell type in the developing cortex. These cells are highly polarized, with a long basal process spanning the entire thickness of the cortex and acting as a support for neuronal migration. The RG cell terminates by an endfoot that contacts the pial (basal) surface. A shorter apical process also terminates with an endfoot that faces the ventricle, with a primary cilium protruding in the cerebrospinal fluid. These cell domains have particular subcellular compositions that are critical for the correct functioning of RG. When altered, this can affect proper development of the cortex, ultimately leading to cortical malformations, associated with different pathological outcomes. In this review, we focus on the current knowledge concerning the cell biology of these bipolar stem cells and discuss the role of their polarity in health and disease.

## 1 Introduction

The cerebral cortex is in the outermost region of the brain and it is responsible in human for high cognitive functions such as problem solving, flexibility, speaking, perception and taking decisions. Corticogenesis is a term that refers to the processes of proliferation, migration, differentiation and synaptogenesis by which the cerebral cortex is formed in mammals, during the development of the central nervous system (CNS). Once formed, it is composed of six distinct neuronal layers. The first step in neurodevelopment is neural tube closure, which takes place at embryonic day 9 (E9) in mice, or gestational week 6 in humans (GW6). The neural tube is a pseudostratified epithelium composed of neuroepithelial cells (NECs) that are highly polarized along the apico-basal axis. This pool of progenitor cells will be amplified by several rounds of symmetric divisions (Dwyer et al., 2016; Götz and Huttner, 2005).

**FIGURE 1:**
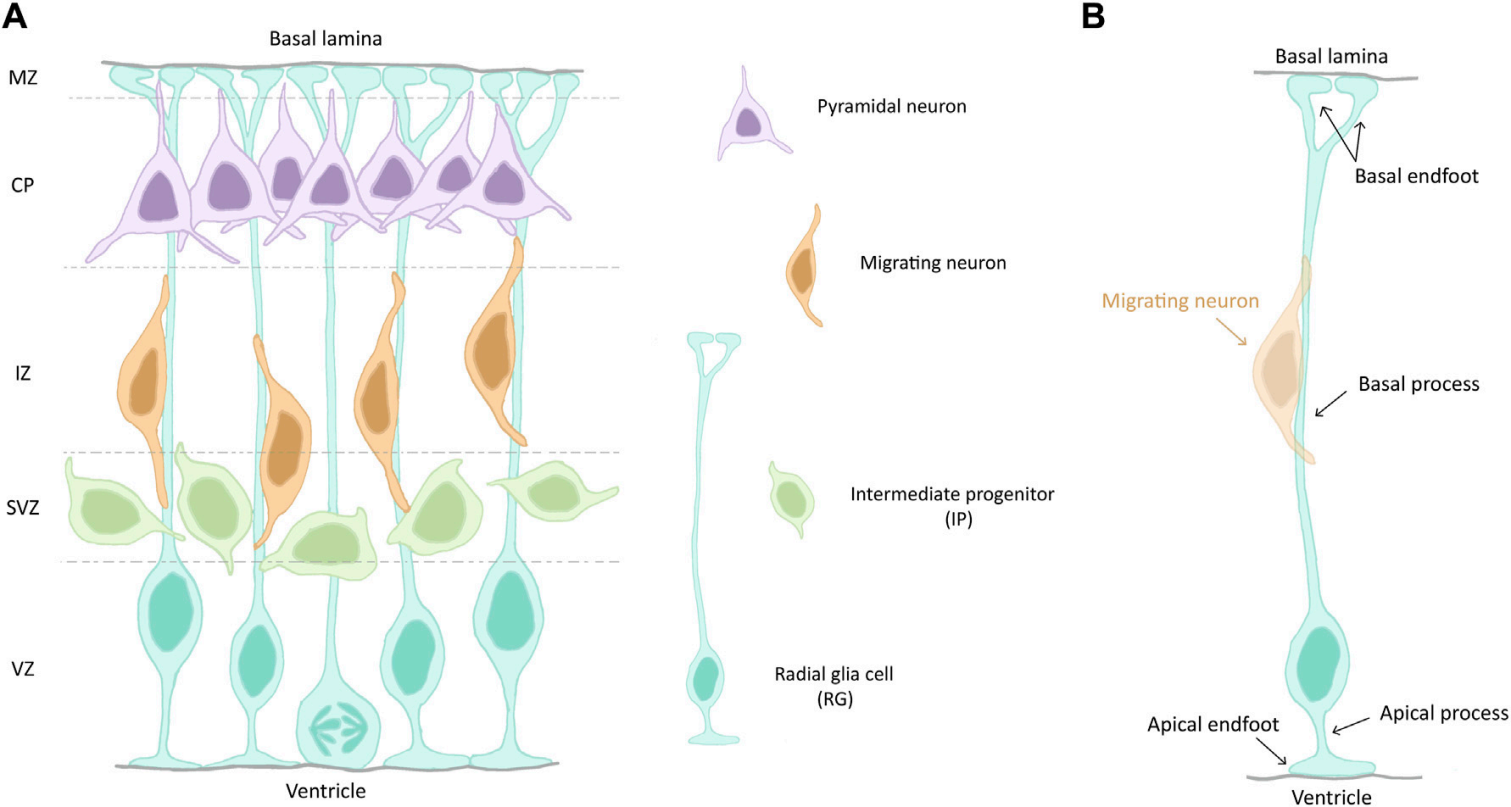
**(A)** Schematic of a section of the mouse developing cortex. Cortical zones are indicated on the left and separated by dashed lines. The different cell types are depicted on the right. **(B)** Illustration of a radial glia cell (RG), highlighting its different cell compartments and features. A migrating neuron (orange) is shown. Abbreviations: VZ, ventricular zone; SVZ, subventricular zone; IZ, intermediate zone; CP, cortical plate; MZ, marginal zone.

With the onset of neurogenesis at E11 (GW8), NECs give rise to more fate-restricted progenitors termed radial glia (RG, Figure 1), a distinct but related cell type, exhibiting both neuroepithelial and astroglial properties (Götz and Huttner, 2005). These apical cells can self-amplify through symmetric division to expand the pool of existing progenitors, or give rise to intermediate progenitors (IPs) or neurons through asymmetric division (Götz and Huttner, 2005). IPs are not attached to the VZ, they are more basal and form the subventricular zone (SVZ). RG can also produce basal RG (bRG). These latter cells are less numerous in mouse compared to primates and are known for their neurogenic potential (Penisson et al., 2019). The earliest born neurons appear at E11 and form the preplate (PP). With the formation of the cortical plate (CP) around E13 (GW9), the PP is divided into the subplate (SP) and the marginal zone (MZ). After this splitting, later born neurons migrate past earlier born neurons, thus constituting the upper layers (L2-L4) and the deep layers (L5 and L6) of the CP, respectively. This temporal sequence of neuronal birth and migration is termed “inside-out” development of the cortex. Neurons migrate radially along RG basal processes to reach their final position in the postnatal and then adult neocortex (Dwyer et al., 2016; Molyneaux et al., 2007) (Figure 1A).

RG are the main progenitor cell type during the development of the cerebral cortex and they are highly polarized cells (Figure 1B). Their cell bodies are restricted to the ventricular zone (VZ), the most apical cell layer that faces the ventricle during development. They undergo interkinetic nuclear migration (INM) during their cell cycle, meaning that their nuclei migrate up and down along the apico-basal axis of the VZ. They are in S-phase when their nuclei are on the basal side of the VZ and in mitosis when they are at the apico-basal bordering the ventricle (ventricular surface). RG in interphase have a short apical process, aiding their attachment at the ventricular surface. The apical process terminates with an endfoot which exhibits a primary cilia (PC), protruding into the cerebrospinal fluid (CSF) and acting as a signalling hub. A longer basal process spans the entire thickness of the cortex and acts a support for neuronal migration (Nadarajah and Parnavelas, 2002). It also terminates with an endfoot, contacting the pial surface (Figure 1B).

**FIGURE 2:**
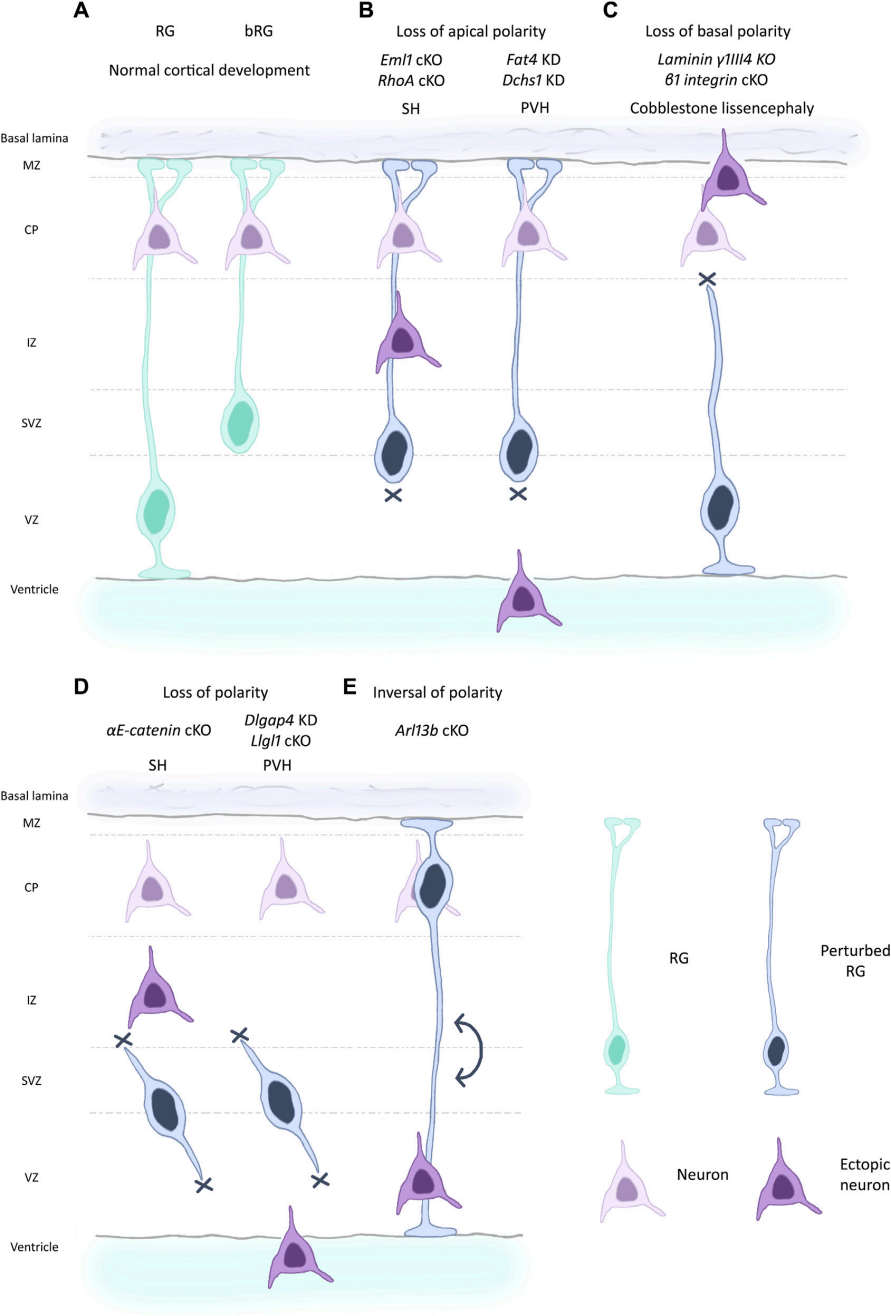
Different scenarios of changed polarity affecting RG and leading to cortical malformations. **(A)** RG and bRG (both light blue-green) are present during normal cortical development and neurons (light purple) are correctly positioned in the cortical plate following migration along the basal process. RG apical detachment giving rise to bRG from RG is regulated by factors such as Plekha7 (Tavano et al., 2018). **(B)** Perturbed RG (dark blue) with loss of apical processes can lead to cortical malformations in the mouse such as subcortical heterotopia (SH, left) e.g., due to mutations in Eml1, RhoA (Zaidi et al., 2024; Cappello et al., 2012). Breakages in the ventricular boundary can also lead to apical cell detachment and periventricular heterotopia (PVH, right) (e.g., mutations in Fat4, Dchs1, Cappello et al., 2013). Ectopic neurons are depicted in dark purple. **(C)** Loss of basal process attachment, often caused by defective signalling, can be accompanied by breaches of the basal lamina. This leads to a cobblestone-like lissencephaly as seen for mutation in laminin and integrin genes, among others (Haubst et al., 2006; Radakovits et al., 2009). **(D)** RG can lose polarity both apically and basally, leading to internalised RG as seen for example, for αE-catenin and Llgl1 mouse models, causing respectively SH (left) and PVH (right)-like phenotypes (Lien et al., 2006; Schmid et al., 2014). **(E)** More rarely, inversion of polarity in RG can be observed as in Arl13b mouse mutants (Higginbotham et al., 2013). In this situation, the cell soma is located next to the basal lamina. Conversely, neurons are found at the ventricular surface.

Establishment and maintenance of RG cell structure and polarity is crucial for their correct functioning, organized neuronal migration, and ultimately for proper cortex development. In this review, we resume the current knowledge on the morphology and cell biology of these bipolar stem cells and discuss the importance of polarity in health and disease. We mention via the study of mutant models, various changes in polarity impacting corticogenesis (Figure 2).

## 2 Asymmetric distribution of organelles

**FIGURE 3:**
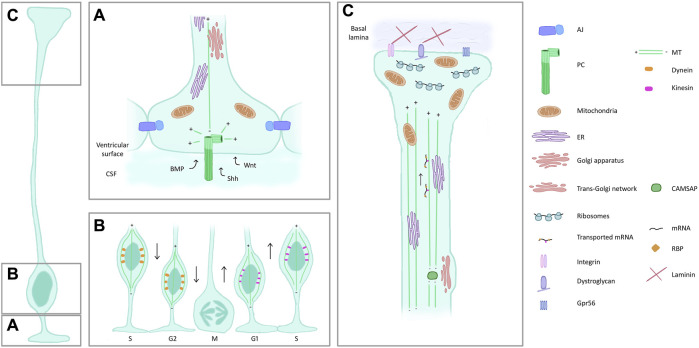
Features and composition of RG compartments. **(A)** Apical process and endfoot. RG are in contact with each other through AJ. A PC (dark green) protrudes in the ventricle, centrioles (light green) act as an MT organizing centre. The PC receives signals from the CSF. Mitochondria, Golgi and ER are also present in the apical side of RG. **(B)** Interkinetic nuclear migration. RG nuclei are found most basally during S phase and move to the ventricular surface to enter mitosis, aided by dynein (orange) along the MT cytoskeleton. The apical to basal movement is supported by kinesin (pink). **(C)** Basal process and endfeet. ER and mitochondria are also found in the basal side of RG, the latter particularly enriched in the endfoot. Trans-Golgi elements are present in basal process varicosities, associated with CAMSAP which acts as an MT nucleator removed from the centrosome. mRNA transport along the basal process and local translation in the basal endfeet are represented. Proteins on the surface of the endfoot (integrins, dystroglycan complex) ensure the contact with the ECM. Abbreviations: AJ, adherens junctions; PC, primary cilia; MT, microtubules; CSF, cerebrospinal fluid; ER, endoplasmic reticulum; RBP, RNA binding protein.

Highly polarized RG show particular intracellular characteristics (Figure 3), of which we cite here a number of examples.

### 2.1 Apical cell-cell adhesion

A key player in apico-basal polarity establishment and maintenance in RG are adherens junctions (AJ). These structures, composed of cadherins and catenins, ensure the cell-cell contacts between the apical membranes of RG and maintain the tissue compact at the ventricular surface (Veeraval et al., 2020) (Figure 3A). AJ recruit polarity proteins, such as Crumbs, Par and Scribble complexes (Jossin, 2020; Singh and Solecki, 2015). These complexes have crucial roles in signalling pathways that help maintain AJ and polarity. Numerous studies show that when these contacts are lost, RG can detach from the ventricular surface with an impact on their polarity, proliferation, and ultimately corticogenesis.

Disrupting apical adhesion components can give rise to periventricular heterotopia (PVH), a phenotype associated with breaks in the ventricular boundary (Klingler et al., 2021; Romero et al., 2018). FAT4 and DCHS1 are protocadherin proteins, respectively receptor and ligand, that are apically located but distinct from AJ and act upstream of the Hippo signalling pathway. Absence of either protein of the pair, through knockdown experiments in mouse, was shown to lead to an accumulation of RG in the SVZ, due to cell detachment, associated with a PVH-like phenotype (Cappello et al., 2013) (Figure 2B, right). Importantly, mutations in *FAT4* and *DCHS1* have been identified in individuals (from four and three families respectively) with Van Maldergem syndrome, an autosomal recessive condition characterized by intellectual disability, craniofacial malformations and PVH (Cappello et al., 2013).

In mouse mutants for αE-catenin, the AJ are heavily disrupted, the ventricular surface is disorganized and RG lose their polarity, with disorganized and almost absent processes (Lien et al., 2006; Schmid et al., 2014). Some internalized rosette structures, where cells maintain contact with each other, were observed (Lien et al., 2006). Dlgap4 is a synaptic scaffolding protein also expressed in RG, and knockdown experiments in the mouse lead to a disrupted ventricular boundary, with reduced expression of actin, catenin and cadherin. RG fibers are also disorganised (Romero et al., 2022). Moreover, the authors identified *DLGAP4* mutations in patients presenting heterotopias and cortical malformations (Romero et al., 2022) (Figure 2D). For additional human genetic information please see Ferent et al., 2020.

Llgl1 is the mammalian ortholog of a *Drosophila* cell polarity gene. This protein makes a link between polarity complexes and AJ (Jossin, 2020; Jossin et al., 2017). Mutations in this gene lead to a phenotype similar to αE-catenin mutants, with RG internalized above the ventricular surface, forming rosettes where the polarity complexes are still detectable and from which RG processes extend outside (Jossin et al., 2017) (Figure 2D).

Another renowned study shows that the AJ specific protein Plekha7 plays a critical role in keeping RG attached at the ventricular surface, as shown by inactivation experiments that lead to RG delamination (Tavano et al., 2018). This is most likely due to Plekha7 interaction with proteins (e.g., of the nectin system and CAMSAPs) that make a link with the cytoskeleton, as suggested by the authors in the discussion. Physiologically, a timely regulated repression of Plekha7 by the transcription factor Insm1 is crucial for delamination of RG to give rise to more basally localized progenitors (Tavano et al., 2018) (Figure 2A, right).

Thus, these examples emphasize how the regulation of AJ complexes and apical adhesion is associated with forming and maintaining RG morphology and polarity.

### 2.2 Centrosomes and primary cilia

Other apical structures such as the centrosome and PC assist in establishing polarity [e.g., see Francis and Cappello (2021) for review]. The PC is an antenna-like structure that acts as a signalling hub by protruding in the CSF to capture signals, and the centrosome is crucial for its formation [reviewed in Zaidi et al. (2022)] (Figure 3A). Defects in these organelles can impact RG polarity and lead to abnormalities in cortical development.

Showing the major role of PC in maintaining RG polarity, deletion of Arl13b, a cilia-specific small GTPase, in mouse cortical progenitors led to a reversal of RG apico-basal polarity and abnormal neuronal positioning (Higginbotham et al., 2013) (Figure 2E). This is likely to be due to improper receptor localization at the PC, impacting downstream signalling, as shown for the IgfR1 receptor (Higginbotham et al., 2013). Mutations in ARL13B are also linked with Joubert syndrome, where patients present cortical malformations and intellectual disability.

Defective centrosomes were observed in mice mutants for Eml1, a microtubule (MT) associated protein (Zaidi et al., 2024). This mutation is associated with apical RG cell detachment leading to subcortical heterotopia (SH) (Figure 2B, left), a cortical malformation characterized by large clusters of neurons in the white matter. In this study, in accordance with other works on the same mutation both in mouse and human models, defects were revealed in the PC as well, which were shorter due to the mutation (Jabali et al., 2022; Uzquiano et al., 2019; Zaidi et al., 2024). The centrosome and PC defects were partially rescued upon Epothilone D (EpoD) treatment (Jabali et al., 2022; Zaidi et al., 2024), an MT polymerizing and stabilizing agent. Mutations in *EML1* are found in patients from eight families, who display SH, epilepsy and intellectual disability (Markus et al., 2021).

Furthermore, upon conditional loss of the centriolar protein SAS4, RG lose their attachment in the VZ, move away and ultimately die, leading to microcephaly in mice, as observed in patients with mutations for *SAS4* (Insolera et al., 2014). Mutant cells lose their centrosome and PC, highlighting their role in RG positioning. CEP83 is also involved in anchorage of centrosomes to the apical membrane. When the gene is mutated, the organization of MTs at the apical surface is affected, possibly altering the mechanical properties of the membrane that becomes wider and more stretched (Shao et al., 2020). Intellectual disability and occasionally hydrocephalus are observed in patients with mutant *CEP83* (Failler et al., 2014).

Thus, we cite examples showing that these linked apical organelles are crucial for RG integrity.

### 2.3 Golgi apparatus

The Golgi apparatus receives, modifies and sorts proteins and lipids to different cell compartments and it is therefore crucial for membrane trafficking (Ravichandran et al., 2020). This will ultimately play a role in cell polarity, as different and specialized regions of the cells require specific lipid and protein compositions.

In RG, it has been shown that the Golgi apparatus is confined in the apical process and is not generally in close proximity with the centrosomes (Taverna et al., 2016). Post-Golgi secretory transport of vesicles was shown to be important in apical processes [(Brault et al., 2022), see also Cytoskeleton section] (Figure 3A). The Golgi apparatus was found to be absent in the basal process, whereas the endoplasmic reticulum (ER) can be found throughout the RG cell (Rash et al., 2018; Taverna et al., 2016) (Figures 3A, C). A later study (Coquand et al., 2021) identified secretory machinery resembling trans-Golgi elements in varicosities of the basal process, playing also a role in MT nucleation (Figure 3C) Nevertheless, cis and medial Golgi elements were not identified, confirming the findings of Taverna et al. In basal progenitors that are not attached apically, the Golgi apparatus becomes associated with the centrosome.

Golgipathies have been linked to microcephaly (presumably affecting RG), and can also involve PC defects [see Passemard et al. (2019); Masson and ElGhouzzi (2022) for further details]. Also, in a model of aberrant RG apical detachment linked to heterotopia (Uzquiano et al., 2019), VZ RG showed abnormal Golgi apparatuses, such as a lower number of Golgi elements and reduced extension of the organelle within the apical process. Golgi anterograde trafficking was shown to be affected. This suggests that changes in polarity (here loss of apical processes), in healthy or pathological conditions, can lead to (or be caused by) reorganization of the Golgi apparatus.

### 2.4 Mitochondria

Mitochondria are key organelles for the proper functioning and survival of a cell. Mitochondria are found in RG cell soma as well as both apical and basal processes and interestingly they seem to be enriched in endfeet (Rash et al., 2018) (Figures 3A, C). A study performed on *Xenopus* neural progenitors showed mitochondria distributed all over the cell. However, they also seemed to be asymmetrically distributed in dividing cells around the cell soma and this, together with mitochondrial remodelling, is likely to be linked to cell fate in multiple organisms (Feng et al., 2023; Iwata et al., 2020; Iwata and Vanderhaeghen, 2021).

Mitochondria transport has been observed along RG processes in organotypic brain slices and this transport is likely to be Ca^2+^ dependent, indeed local calcium release slows mitochondrial movement (Rash et al., 2018). This team had observed previously that Ca^2+^ is able to propagate bidirectionally through RG processes, its source residing in the ER, and this is particularly high in RG endfeet (Rash et al., 2016), possibly explaining mitochondria enrichment at this location. In hyperglycaemic conditions, that affect glucose metabolism and therefore mitochondria, RG processes collapse, and slower mitochondria transport is observed (Rash et al., 2018). Whether this could be at the origin of any cortical malformation is not yet known, although perhaps likely.

A recent study on the human-specific protein ARHGAP11B described its role in mitochondria (negative regulation of membrane permeability), and notably it regulates the transition of apical to basal RG by stimulating glutaminolysis (Xing et al., 2024).

These results show how mitochondria localization, distribution and function in apical RG help maintain the bi-polarity of these cells.

### 2.5 Basal process: inheritance and cell fate

The basal process protruding from the soma of RG spans the entire thickness of the cortex to contact the pial surface. It has the crucial role of providing a scaffold for neuronal migration during corticogenesis [extensively reviewed in (Meyerink et al., 2020)] (Figure 1B). We discuss here specifically its maintenance.

As mentioned in the introduction, RG can divide through asymmetric division to give rise to a daughter RG together with an IP, bRG, or a neuron. How basal process inheritance plays a role in cell fate outcome has been the subject of several debates.

A pioneer study showed that the daughter neuron inherits the basal process, while the progenitor will regrow a new one (Miyata et al., 2001), but the consensus is now that the basal process is largely inherited by the progenitor daughter cells (Alexandre et al., 2010; Konno et al., 2008; Tsunekawa et al., 2012). Inheritance of both apical and basal processes is hypothesized to be important for self-renewal capabilities (Konno et al., 2008). CyclinD2, localized in the basal endfoot of RG, is asymmetrically inherited by the most basal daughter cell and will dictate self-renewing fate. Overexpression and knockdown experiments, altering asymmetric distribution of CyclinD2, perturb RG cell fate output (Tsunekawa et al., 2012). Live imaging in zebrafish neural tube also showed that the most basal daughter cell inherits the basal process and commits to progenitor fate (Alexandre et al., 2010).

The basal process is also inherited by proliferating bRG, often originally generated through oblique cell division (Penisson et al., 2019; Shitamukai et al., 2011, see also Cytoskeleton section). Therefore, is clear that the basal process plays a key role in RG polarity (see also Extracellular signals section).

## 3 Cytoskeleton

The cytoskeleton is crucial for maintenance of the structure and morphology of RG while providing the support for trafficking of organelles and proteins that help to establish polarity.

### 3.1 Maintenance of RG structure

The cytoskeleton is composed of intermediate filaments, actin filaments and MTs and is critical for RG structure. For example, treatment of the RG-like cell line C6-R with drugs such as nocodazole and taxol, disrupting MT dynamics, leads to the alteration of their bipolar morphology with cells losing their processes, showing the crucial importance of these components (Li et al., 2003). We also cite here examples of specific proteins influencing different aspects of the cytoskeleton.

The Lis1-Nde1 complex stabilizes the dystrophin/dystroglycan glycoprotein complex (DGC), allowing the formation of a multi-protein complex that links the actin and MT cytoskeletons of RG to the extracellular matrix (ECM), helping with the maintenance of radial morphology and cell-cell adhesion (see also Extracellular components section). Lis1-Nde1 mutations were found to cause deformed and disjointed RG that impaired self-renewal and neuronal migration as a consequence. Functional insufficiencies of *LIS1, NDE1* and dystroglycan are all known to cause lissencephaly syndromes in patients (Pawlisz and Feng, 2011). Deletion of Eml1, mentioned above, also affects MT growth and dynamics, with partial rescue of the resulting SH phenotype achieved upon treatment with Epothilone D (Zaidi et al., 2024), which also rescued centrosome and PC phenotypes (Jabali et al., 2022; Zaidi et al., 2024).

Related to the actin cytoskeleton, as stated previously, deletion of αE-catenin in the developing mouse cortex leads to severe disruption of RG polarity and subsequently to the formation of SH, and this is caused by the uncoupling of AJ with intracellular actin fibres, leading to an increased subcellular G-actin/F-actin ratio (Schmid et al., 2014). Deletion of the small GTPase RhoA leads to the migrational disorders of SH and cobblestone lissencephaly (Figure 2B, left and 2C) as a result of a defective RG scaffold, disrupted upon destabilization of both the actin and MT cytoskeletons (Cappello et al., 2012). Other actin modulator Rho-GTPases, Cdc42 and Rac1, were also shown to affect RG morphology when mutated (Cappello et al., 2006; Leone et al., 2010; Yokota et al., 2010). Furthermore, mTOR signaling, which is associated with several neurodevelopmental disorders, is found to regulate basal RG morphology and neuronal migration by modulating Rho-GTPase-mediated organization of the actin cytoskeleton (Andrews et al., 2020). Dlgap4, mentioned above, also impacts actin cytoskeleton dynamics, affecting RG morphology and causing a ventricular surface (PVH) phenotype in mouse and SH in human (Romero et al., 2022) (Figure 2D, right).

Nestin, vimentin and GFAP are well known intermediate filament markers for glia, including RG (de Reus et al., 2024). RG are likely to also strictly require these less well-studied structural proteins (Li et al., 2021), potentially aiding organelle movement and distribution.

### 3.2 Interkinetic nuclear migration (INM)

Apart from the maintenance of RG morphology and scaffolding, the cytoskeleton also plays an important role in the process of INM. For completeness in this review, we mention this crucial RG process.

During cell cycle progression, the nuclei of apical RG move between apical and basal sides of the VZ. The nuclei move away from the apical surface towards the basal side during G1 phase, undergo S phase at the basal position, and return towards the apical side during G2 phase for mitosis (Figure 3B). INM in mammalian apical RG is mediated by MT-based processes, and the apical to basal movement is driven at least in part by the actin-myosin system and displacement by active apical nuclear movement (Kosodo et al., 2011; Schenk et al., 2009; Spear and Erickson, 2012; Tsai et al., 2010). MTs and the minus end directed motor protein dynein are important for the basal to apical movement and kinesin for basally directed nuclear movement (Tsai et al., 2010) (Figure 3B). Concerning daughter cells, the one that inherits the basal process (committed to progenitor fate, as described in Section 1) will move the nucleus more quickly away from the apical region compared to sibling cells generated in a morphologically unpolarized manner, helping to avoid overcrowding during INM. Indeed, removal of the basal process by inhibition of TAG-1, a glycoprotein involved in adhesion (see also Extracellular section below), results in abnormally highly-packed progenitors apically, which will detach and lead to heterotopia (Okamoto et al., 2013). Daughter cell polarity hence contributes to correct corticogenesis.

A number of MT or MT motor associated proteins when impaired lead to a disrupted INM in the neocortex of rodents. Disruption of factors that impact the organization and integrity of MT such as CEP120, TACCs, Hook3, PCM1 and TPX2, were found to impact INM (Ge et al., 2010; Kosodo et al., 2011; Xie et al., 2007).

Furthermore, the mutation of dynein regulators such as Lis1 and NudC also impact this process (Cappello et al., 2011; Tsai et al., 2005). Blebbistatin inhibition of non-muscle myosin II at low concentrations to selectively inhibit the INM in RG while maintaining the structural integrity in slice cultures revealed that there was selective impairment of apical to basal nuclear migration. Indeed, this movement requires myosin II mediated constriction of the apical process which pushes the nucleus in the basal direction (Schenk et al., 2009). In addition, inhibition of the PITP/ncPCP- signaling pathway is found to impair INM and in turn tangential expansion of the cortex by deregulating actomyosin activity in the nuclear periphery of RG (Xie and Bankaitis, 2022).

Thus, multiple pathways are crucial for INM, allowing polarized movements within RG and correct cell cycle.

### 3.3 Mitotic spindle formation

The formation of the oriented mitotic spindle, an MT-based structure in apical RG, ensures proper chromosomal segregation and inheritance of cell fate determinants by controlling the angle of division (di Pietro et al., 2016; Matsuzaki and Shitamukai, 2015). The orientation of the mitotic spindle therefore affects cell lineage specification of the progeny.

While early apical RG predominantly exhibit vertical cleavage plane divisions, conditional deletion of Afadin for example, and overexpression of Inscuteable in mouse are found to increase oblique divisions favoring the production of IPs (Fish et al., 2008; Postiglione et al., 2011; Rakotomamonjy et al., 2017). The bRG cells in human may be increasingly produced by horizontal cleavage plane divisions of the ventricular apical RG [Lamonica et al. (2013), see also Penisson et al. (2019) for further discussion]. Clearly division angles and polarity (choice of apical or basal process inheritance or re-growth) must be linked, although little is known concerning these regulatory steps.

It is known though that genes that are implicated in microcephaly are often involved in centrosome biogenesis and maturation, and/or spindle orientation (Noatynska et al., 2012). Mouse Aspm protein is normally localized at the mitotic spindle poles of NECs and is downregulated upon the switch from proliferative to neurogenic divisions. RNA interference (RNAi) of Aspm leads to changes in the perpendicular orientation of cleavage planes, most probably causing increased asymmetric divisions, favoring thus neurogenic over proliferative divisions (Fish et al., 2006). Human mutant *ASPM* cortical organoids displayed transient randomization of mitotic spindle orientation leading to precocious generation of bRG while depleting the amplification of ventricular apical progenitors (Benthem et al., 2023). Deletion of Mcph1 in mouse led to uncoupling of mitosis and the centrosomal cycle causing premature mitotic entry, upon Chk1 not localizing to the centrosome. This led to a shift in the alignment of the mitotic spindle favoring neurogenic cell fate over the proliferation of progenitors (Gruber et al., 2011). Similarly, deletion of factors which are important for centrosomes such as CDK5RAP2, CPAP, STIL and CEP63 also led to spindle orientation defects (Garcez et al., 2015; Kitagawa et al., 2011; Lizarraga et al., 2010; Marjanović et al., 2015).

Deletion of the lissencephaly gene Lis1 results in less stable astral MTs and causes defects in mitotic spindle positioning, increasing premature asymmetric neurogenic divisions and reducing the cell number (Yingling et al., 2008). Mutations in Lis1 related proteins such as Magoh, Dcx and NdeI also result in spindle orientation defects (Feng and Walsh, 2004; Pramparo et al., 2010; Silver et al., 2010). Mitotic spindle lengths were also found to be abnormally long in the apical progenitors of *Eml1* mutant mice which exhibit excessive RG delamination (Bizzotto et al., 2017). It is possible in this case that mechanical forces are changed in the VZ, consequently altering apical RG attachment.

### 3.4 Intracellular trafficking

The MT cytoskeleton is important for the polarized transport of cargoes to the apical and basal ends of RG (Wimmer and Baffet, 2023). Subcellular live imaging of mouse brain tissue revealed that most of the MTs in the apical process emanated from the pericentrosomal region with an apical to basal direction (Figure 3A). On the other hand, MTs in the basal fibres of apical RG and human basal RG were oriented in both directions (however with a basal bias) emanating from the acentrosomal MT organizing centres localized in varicosities of the basal fibre (mentioned above), dependent on the CAMSAP family of proteins, in addition to those emanating from the centrosome (Coquand et al., 2021) (Figure 3C).

Memo1, critical for RG tiling (non-randomly arranged and regularly interspaced RG basal processes) during neocortical development, was found to regulate MT stability and dynamics of the basal process. Deficiency of Memo1 led to disrupted CAMSAP2 distribution at MT minus ends leading to aberrant branching of MTs and alteration of polarized trafficking of the basal domain protein Gpr56 (Nakagawa et al., 2019) (see also section Local translation).

The apical MT network in RG helps with the transport of cargoes from the Golgi apparatus to the apical surface via dynein-based transport mechanisms. The apical post-Golgi transport of Crumbs via Rab6+ vesicles was shown to be important for apical polarity complexes and the maintenance of apical junctions. Deletion of the dynein activator and lissencephaly gene Lis1, or Rab6, disrupts this transport leading to the loss of apical AJ and cell delamination (Brault et al., 2022).

Mutations in *ARFGEF2* are associated with microcephaly and PVH in patients. Inhibition of the ARFGEF2 encoded protein BIG2 in MDCK cells led to the disruption of trafficking of E-cadherin and β-catenin from the Golgi apparatus to the cell surface, showing that vesicular trafficking is important for normal human cerebral cortical development (Sheen et al., 2004). It seems likely that apical trafficking in RG may be disrupted explaining the PVH phenotype. As mentioned above, Eml1 loss of function in mouse impaired post-Golgi vesicular trafficking, including of selected PC proteins such as SSTR3 and PKD2, which will have an impact on RG structure and function (Uzquiano et al., 2019; Zaidi et al., 2024). Thus, there are multiple examples suggesting a link between intracellular trafficking and RG morphology, attachment, polarity and corticogenesis (see also below Local translation section).

## 4 Extracellular components in the generation and maintenance of polarity

Apart from intracellular factors required for the formation and maintenance of RG polarity, it is important to mention extracellular factors which also contribute to these processes, impacting RG structure and morphology. Owing to the presence of their apical and basal processes terminating in the CSF and at the pial surface respectively, as well as extracellular factors across whole RG cell surfaces, these cells can receive many signals [for review see (Ferent et al., 2020)].

### 4.1 Signalling factors influencing RG structure

RG structure is clearly influenced from signals received in the CSF, e.g., through PC (Figure 3A). In addition, cell-cell and cell-environment contribute in shaping their structure.

Growth factors such as FGF2, EGF, IGF, BDNF and TGF-β1 were shown to influence the proliferation and maintenance of RG (Bartkowska et al., 2007; Kang et al., 2009; Lamus et al., 2020; Raballo et al., 2000; Stipursky et al., 2015; Zappaterra and Lehtinen, 2012). As an example, injection of TGF-β1 into the embryonic ventricles at E14 led to notably disorganized RG fibres (Stipursky et al., 2015).

Other secreted factors from distant sources e.g., found in embryonic CSF, such as Bmp, Wnt, Shh, and from more local sources e.g., nearby cells producing for example, Neuregulins, Retinoic acid and Reelin, can also influence RG behaviour and maintenance [see (Ferent et al., 2020) for further details]. Glial growth factor secreted by neurons migrating along RG fibres was shown to positively influence the growth of the RG fibre which is critical for neuronal migration (Anton et al., 1997).

Biallelic missense mutations in endothelin converting enzyme-2 (ECE2) have been found to be associated with PVH in human (Buchsbaum et al., 2020). Knockdown and overexpression of ECE2/Ece2 in human cortical organoids and developing mouse tissue led to changes in the bipolar morphology of RG and the mispositioning of ectopic neurons in the VZ. Proteomic analyses of ECE2 KO human cortical organoids revealed downregulation of ECM components and receptors such as laminins, lumican, decorin and six different collagens (Buchsbaum et al., 2020).

Cajal-Retzius neurons are found in the most superficial layer of the developing cortex, in the MZ. Reelin, a glycoprotein secreted from Cajal-Retzius cells was shown to influence apical-basal radial processes (Hartfuss et al., 2003; Supèr et al., 2000; Zhao et al., 2004) and branching of the basal processes (Chai et al., 2015). Other secreted factors from Cajal-Retzius cells almost certainly also influence these processes [e.g., see (Borello and Pierani, 2010)].

BDNF is known to influence the growth of the basal process and therefore the RG scaffold by activating a Ca2+ activated chloride channel Anoctamin 1. Lack of radial process extension in Ano1-KO mice leads to disorganization of cortical layers and significantly reduced cortical thickness (Hong et al., 2019).

### 4.2 Contact with the meninges: maintenance of polarity and cell survival

Expression of ECM components identified through several mouse and human transcriptome and proteome analyses, such as laminins, proteoglycans, dystroglycans and collagens (also mentioned above) were shown to influence the proliferation of apical and basal progenitors (Buchsbaum et al., 2020; Fietz et al., 2012; Florio et al., 2015; Kalebic and Huttner, 2020; Pollen et al., 2015). In addition, they also play a role in the formation and maintenance of the RG scaffold. Indeed, the basal process terminates with an endfoot that contacts the meningeal basement membrane (BM) and these contacts are mediated by the above mentioned proteins (Graus-Porta et al., 2001; Haubst et al., 2006; Myshrall et al., 2012; Radakovits et al., 2009) (Figure 3C).

Laminin is important for maintaining the structural integrity of the BM. Mutations in Laminin beta-1 (LAMB1) lead to cobblestone-lissencephaly in patients, caused by over-migration of neurons upon detachment of the basal endfeet of RG (Radmanesh et al., 2013). Similarly, targeted deletion of the nidogen-binding site within the laminin γ1 chain and deletion of perlecan in mice also lead to disruption of the BM and formation of neuronal ectopias that resemble the cobblestone-lissencephaly phenotype (Haubst et al., 2006) (Figure 2C).

Upon loss of β1 integrin, basal endfeet lose their anchoring to the BM and RG fibres appear irregular (Graus-Porta et al., 2001; Haubst et al., 2006; Radakovits et al., 2009). Moreover, in β1 integrin mutants this is followed by RG death, as observed with apoptotic markers as well as live imaging. These phenotypes are undetectable after E15, suggesting that signals from the meninges are crucial for RG survival, particularly in early development, which ultimately affects proper cortex development (Haubst et al., 2006; Radakovits et al., 2009). Later on, attachment to the BM becomes crucial for RG integrity and neuronal composition as observed in a laminin mutant where GABAergic interneurons populate the outer regions of the cortical plate, where Math2+ pyramidal neurons are not detected (Haubst et al., 2006; Myshrall et al., 2012).

Examples of signals downstream of β1 integrin include kinases MAPK, shown to influence cell survival of neural stem cells in culture (Campos et al., 2004), and ILK, that has been shown to regulate neuronal polarity, as its inhibition led to perturbed axon formation, but did not affect dendrites (Niewmierzycka et al., 2005). FAK, a non-receptor tyrosine kinase, also regulates cell growth and survival, and its deletion in the mouse brain leads to perturbed basal endfeet, either unattached to the BM or protruding into neuronal ectopias resembling cobblestone lissencephaly (Beggs et al., 2003). Blocking the fixation of laminin to its ligand β1 integrin in mouse cerebral cortex also led to the detachment of RG apical processes, suggesting the similar importance of the laminin-integrin interaction for the maintenance of apical process (Loulier et al., 2009).

Dystroglycan, another ECM component and a cell surface laminin receptor protein, is essential for the maintenance of BM integrity (Henry and Campbell, 1998). Conditional inactivation of Dag-1 encoding dystroglycan in mouse embryonic cortex led to pial BM disruption and formation of neuronal ectopias in the meninges (Myshrall et al., 2012). Similarly, patients showing defective O-glycosylation of α-dystroglycan display several brain abnormalities including neuronal over-migration causing a cobblestone cortex (Van Reeuwijk et al., 2005) (Figure 2C).

TAG1/contactin-2 (Transient axonal glycoprotein-1), mentioned previously, is a cell surface molecule expressed in the basal region of the cortical wall. It is important for the maintenance of RG basal processes and knockdown of TAG1 was shown to cause basal process retraction and ectopic progenitors in mouse (Okamoto et al., 2013). Activation of Notch was also shown to promote radial morphology of RG clones through increased expression of BLBP and the cell adhesion molecule nidogen, which binds to laminin (Li H. et al., 2008).

Finally, deletion of Gpr56, a GTPase expressed in basal endfeet, leads to disruption of the BM and endfeet breakout through the broken meninges (Li S. et al., 2008), suggesting that it might regulate proper endfeet anchorage at the pial surface. GPR56 mutations in human give rise to cobblestone lissencephaly and polymicrogyria (Jaglin and Chelly, 2009) (Figure 2C). Furthermore, with the inhibition of Follistatin like-1, a secreted glycoprotein from the pial BM, the RG basal processes were no longer found to be parallel to each other and their basal endfeet exhibited greater density and branching (Liu et al., 2015). For further human genetic information please see Ferent et al. (2020).

Thus, pial surface interactions are critical for retaining RG morphology and function, influencing brain development. These multiple examples show the importance of short and long-distance extracellular molecules influencing RG structure and polarity.

## 5 Local translation at distal sites of RG

mRNA localization and local translation are key mechanisms that a polarized cell requires to quickly reply to local stimuli acting far away from the soma. This has been thoroughly studied in highly polarized cell types such as neurons and astrocytes (Holt et al., 2019; Mazare et al., 2021).

Recent evidence is emerging supporting a role of local translation in RG as well. Using live imaging in organotypic mouse brain slices, Pilaz et al. observed mRNA and RNA binding proteins (RBP) moving along the basal process of RG, in a MT-dependent fashion (Figure 3C). After microdissection of RG basal endfeet, the authors used photoconvertible proteins to demonstrate local translation: these protein constructs bear a fluorescent tag that change colour upon exposure to UV light (e.g., green to red). Recovery of green fluorescence demonstrated local translation of several mRNAs (*Ccnd2*, *Kif26a*). A set of FMRP-bound transcripts was also identified in these microdissected regions (Pilaz et al., 2016), likely to have been transported along the basal process. In a following study by the same authors, *Arghap11a* was also shown to be localized and translated basally, this being crucial for basal endfeet morphology (Pilaz et al., 2023).

More recently, a basal endfeet proteome was obtained through *in vivo* proximity labelling, and identified proteins specifically enriched in basal endfeet (e.g., Myh9 and Myh10), as well as their transcripts, suggesting that they might also be locally translated. Loss of these basal proteins leads to a reduction in endfeet branching and their protrusion through the BM (MYH9), as well as loss of apical and basal attachment (MYH10) (D’Arcy et al., 2023). Overall this shows how local translation can be critical to dictate and maintain RG structure and polarity.

Local translation in apical endfeet has not yet been demonstrated. Nevertheless, it is known that some transcripts and proteins are apically enriched, suggesting that these might be locally translated as well. The RNA-binding protein Staufen2 (Stau2), known to regulate asymmetric RNA localization in *Drosophila* neuroblasts (Chia et al., 2008) is also asymmetrically distributed in RG, being enriched apically in a complex with Pumillio2 (Pum2), a translational repressor, and Ddx1, an RNA helicase. *β-actin* and *Prox1* mRNAs were found associated with Stau2 from E12.5 brain cortices and were shown to be localized apically. Knockdown of either of the proteins in the complex leads to mislocalization of *Prox1* mRNA and results in increased neurogenesis and reduction of the RG pool. Interestingly, Prox1 protein expression increases upon Stau2 knockdown, suggesting that the mRNA is translationally repressed in the complex and this helps maintain the RG in a precursor state. Moreover, Stau2 segregates asymmetrically in cells in mitosis and accumulates, with its cargo RNAs, in the daughter cell that will later give rise to Tbr2+ IPs (Kusek et al., 2012; Vessey et al., 2012). More RNAs are likely to be involved in this process as suggested by Kusek et al. (2012). A set of RNAs identified by Stau2-RNA immunoprecipitation are already known to play a role in cell fate decision, such as *Hes6*, *Cdk5* and *Insm1* (known to influence cell adhesion, see Asymmetric distribution section). Another subset of identified mRNAs code for proteins of the Bardet-Biedl Syndrome complex, an apical complex associated with centrosomes and crucial for PC formation. As mentioned previously, dysfunctioning of PC can lead to SH (e.g., via apical process detachment) and overexpression of Stau2 causes PVH, possibly linking these two observations (Kusek et al., 2012).

Recently, a neural specific centrosome proteome was obtained (O’Neill et al., 2022). RNA binding and RNA processing proteins were enriched, with factors involved in RNA transport and translation regulation. This suggests the possibility of RNA regulation at the centrosome, in accordance with previous studies that identified RNAs at this location (Safieddine et al., 2021; Sepulveda et al., 2018), as well as at the mitotic spindle (Hassine et al., 2020), and their transport in a co-translational dependent manner. RNAs at the RG apical centrosome could be locally translated (Iaconis et al., 2017) and this might have a role in maintaining the growing MT stemming from the MT-organizing centre (MTOC), or perhaps also components of the PC or AJ.

These examples highlight the importance of basally and apically localized proteins and mRNAs in regulating the balance between RG maintenance and differentiation, as well as impacting attachment and structure. Further investigation on apical and basal mRNA and protein localization and translational control is needed.

## 6 Conclusion and perspectives

In this review, we summarize and highlight different factors that influence the polarity of RG. We focus on canonical apical RG exhibiting apical and basal processes. Correct polarity is crucial for proliferation, appropriate neurogenesis and neuronal migration, and any disturbance in these processes lead to cortical malformations as discussed in this review. The unique morphology of RG exposes them to multiple extracellular cues at different levels across the developing neural tissue, in addition to the intracellular factors that define them. Although much knowledge is available in the field of neural progenitor cell polarity, certain interesting understudied topics are gaining more attention and we have attempted to highlight a number of these. For example, the importance of organelles such as mitochondria and the Golgi apparatus, as well as local translation, are emerging as important for RG structure, polarity and function, even if further studies will be necessary to decipher their roles.

Live imaging studies by Rash and colleagues (Rash et al., 2018) as discussed in this review, identified the enrichment of mitochondria at endfeet locations and it would be interesting to know the particular functional relevance of this phenomenon, even if a general disturbance in mitochondrial transport was already associated with collapse of the RG scaffold. It is further interesting to note that studies involving changes in mitochondrial inheritance, as well as fission and fusion dynamics in neural progenitor cells were shown to impact cell fate (Feng et al., 2023; Iwata et al., 2020). Further elucidating mitochondrial and other organelle states, distribution and roles could shed light on the regulation of polarity.

It is an interesting finding that the Golgi apparatus is confined to the apical process of RG but not the basal process (Taverna et al., 2016). Post-Golgi trafficking has been shown to be crucial for maintenance of apical polarity complexes and junctions (Brault et al., 2022), and defects in post-Golgi trafficking were also found in *Eml1* mutant situations leading to SH (Uzquiano et al., 2019; Zaidi et al., 2024). This paves the way for much needed investigations of Golgi position and post-Golgi trafficking defects in RG in different cortical malformations to understand the importance of this polarized localisation. Trans-Golgi network outposts in varicosities (Coquand et al., 2021) are nevertheless required in basal processes. This remarkable adaptation to suit RG morphology and function requires further fine exploration.

Although local translation is a well-studied phenomenon in neurons and astrocytes, local translation in polarized RG has gained attention only recently. Indeed, it can be expected that there might be local translation at RG extremities of the known apically and basally located proteins. Basal endfeet transcriptome and proteome studies confirmed the occurrence and importance of local translation in the basal endfeet (D’Arcy et al., 2023; Pilaz et al., 2016; 2023). Also, apically localized mRNAs have been identified in the apical endfeet and near the centrosomes of RG (Kusek et al., 2012; O’Neill et al., 2022; Vessey et al., 2012) suggesting the occurrence of local translation. Overcoming the technical challenges of apical endfeet isolation in the future will firmly show the importance of such a phenomenon in these cell compartments. Local translation could also occur in regions surrounding the centrosomes, and locally generated proteins could then have multiple roles in apical processes. Perturbations of local translation are expected to greatly impact polarity.

We describe here multiple changes impacting bipolar RG. It is clear that resulting changed cells in some cases may still exhibit polarity (for example, apical RG converted into basal RG often showing a monopolar form) (discussed also by Kalebic and Namba, 2021). In physiological situations, multiple RG states are indeed likely to exist, including SNPs, truncated RG and SAPs [not mentioned here, Arai and Taverna (2017); Nowakowski et al. (2016); Pilz et al. (2013)]. In mutant situations, changes can be dramatic, potentially impacting both apical and basal processes. Multiple pathways may impact attachment, changing cell position and/or morphology. Apical detachment could also influence basal attachment (and *vice versa*), although this is not well understood. It is clear though that for apical RG, remaining attached is important for retaining correct polarization and function.
